# Light and scanning electron microscopy of the eye of *Siganus luridus* (Rüppell, 1828)

**DOI:** 10.3389/fvets.2024.1417278

**Published:** 2024-09-20

**Authors:** Amira Derbalah, Samir A. A. El-Gendy, Hanan H. Abd-Elhafeez, Soha Soliman, Ahmed A. El-Mansi, Manal Seif, Ahmed M. Rashwan, Mamdouh B. Eldesoqui, Catrin Sian Rutland, Valentina Kubale, Mohamed A. M. Alsafy

**Affiliations:** ^1^Histology and Cytology Department, Faculty of Veterinary Medicine, Alexandria University, Alexandria, Egypt; ^2^Anatomy and Embryology Department, Faculty of Veterinary Medicine, Alexandria University, Alexandria, Egypt; ^3^Department of Cell and Tissues, Faculty of Veterinary Medicine, Assiut University, Assiut, Egypt; ^4^Department of Histology, Faculty of Veterinary Medicine, South Valley University, Qena, Egypt; ^5^Biology Department, College of Science, King Khalid University, Abha, Saudi Arabia; ^6^Anatomy and Embryology Department, Faculty of Veterinary Medicine, Matrouh University, Mersa Matrouh, Egypt; ^7^Department of Anatomy and Embryology, Faculty of Veterinary Medicine, Damanhour University, Damanhour, Egypt; ^8^Department of Life Science Frontiers, Center for iPS Cell Research and Application, Kyoto University, Kyoto, Japan; ^9^Department of Basic Medical Sciences, College of Medicine, AlMaarefa University, Riyadh, Saudi Arabia; ^10^School of Veterinary Medicine and Science, University of Nottingham, Nottingham, United Kingdom; ^11^Institute of Preclinical Sciences, Veterinary Faculty, University of Ljubljana, Ljubljana, Slovenia

**Keywords:** *Siganus luridus*, eye, orbit, lens fibers, photoreceptors

## Abstract

**Introduction:**

The morphological characteristics of eyes in fishes are highly diverse and have evolved to meet the specific visual requirements as per their habitats. These morphological features of eyes are important for researchers and ecologists. The dusky spinefoot (*Siganus luridus*) is a tropical teleost fish with a laterally flattened body which lives in the Mediterranean Sea. Currently, there are no histological data relating to the *Siganus luridus* eye.

**Methods:**

In this study, the morphology of the *Siganus luridus* eye was examined to enhance our understanding of its structure and its relationship to fish ecology. Detailed gross and microscopic features were recorded using light and scanning microscopy.

**Results:**

The key observations describe the main structural features of the eye of *Siganus luridus*, specifically, the diameter of the orbit, architecture of three tunics of eye and detailed lens description. The choroid was divided into four layers, and had a *rete mirabile*, consisting of numerous small blood vessels in the choroidal gland. The *tapetum lucidum* was observed, which is interesting since *Siganus luridus* is herbivore and herbivores typically lack a *tapetum lucidum*.

**Discussion:**

These observations shed new light on the intricate eye structure of *Siganus luridus* and provide valuable insights into its visual abilities and adaptations to the aquatic environment and feeding behavior.

## 1 Introduction

The morphological characteristics of eyes in fishes are highly diverse between species and have evolved to meet the specific visual requirements as per their environments and lifestyles. Understanding these characteristics is important for recognizing fish sensory capabilities, behavior, and ecological roles. This knowledge aids in studying fish ecology and evolution within aquatic ecosystems, as well as developing conservation strategies and management practices.

Key morphological features of fish eyes include the position of the eyes, their size, pupil shape, retinal structure, *tapetum lucidum*, color vision, depth perception, and adaptations to the environment (1–3). For example, predatory fish often have forward-facing eyes for binocular vision, while prey species have side-positioned eyes for a broader field of view (4). Nocturnal or deep-sea fishes typically have larger eyes to get more light, and pupil shapes vary to control light intake based on habitat and behavior (5). The retina’s structure, with varying densities of cone and rod cells, determines a fish’s visual abilities, such as color vision and low-light vision. Many fishes possess a reflective layer called the *tapetum lucidum*, enhancing vision in low-light conditions by reflecting light back through the retina (6). The iris itself in marine fish fulfills two purposes: it prevents out-of-focus light from reaching the retina and serves as camouflage (7).

Adaptations in the retinal structure contribute to the fish’s ability to see under different light conditions (8). Many fish have a reflective layer, the *tapetum lucidum*, behind the retina, which improves their vision in low light by reflecting the light that passes through the retina back into the eye, increasing sensitivity in dark environments (8–10). The ability to perceive and distinguish colors varies among fish species, some perceive a broad spectrum of colors, while others have limited color vision (5).

The Dusky spinefoot (*Siganus luridus*), a teleost fish, a tropical or subtropical species, recently migrated into the Eastern Mediterranean sea via the Suez canal. It is a shallow water herbivore that feeds on algae (11, 12) mainly during the day (13). In many teleost fishes, the eyes are located on the sides of the head (14, 15), but some fish species, such as flatfish, have both eyes on the same side of the head (16) or on a dorsal position on the head (17). A wide range of visual adaptations, particularly with regards to retinal cell structures, have been observed in teleosts, primarily related to their lifestyle and visual environment, with the most significant changes in photoreceptor types, arrangements and densities (18–20). Vision in water can be influenced by pressure, feeding habits and temperature (21). Light density was proposed to be the most important physical factor in the evolution of eye shapes (22).

The sclera is the outer fibrous layer that covers most of the eye in the posterior portion, with the cornea located in the anterior portion (23). In most vertebrates, the sclera contains cartilage or bony ossicles to provide more support to the eyeball (24, 25). The cornea of fish has a lower refractive index than the surrounding water medium. Nevertheless, it is a protective covering for the eye and provides an optically smooth surface and a transparent window. Many specializations are known in comparison to the mammalian cornea. These specializations include spectacles and corneal filters (26), iridescent layers (27, 28), and an autochthonous layer (29, 30).

This study aimed to explore the morphology of *Siganus luridus’s* eye to deepen our knowledge of fish eye anatomy and vision, as present no histological data is available in the literature. Studying the anatomy of fish eyes can provide valuable insights into the ecological relationships and adaptations of fish species, gain insights into how fish navigate their surroundings, detect predators or prey, and communicate with other fish and is helping us to better understand and conserve aquatic ecosystems.

## 2 Materials and methods

### 2.1 Specimens and ethics

Ten mature, healthy *Siganus luridus* fish were collected from the Mediterranean Sea (Damietta Governorate). This study was conducted in accordance with the ethical protocols according to Directive 2010/63/EU (2010) 119, in accordance with the ARRIVE guidelines, with ethical approval from the Institutional Animal Care and Use Committee (ALEXU-IACUC; Approval number: 02/13/2022/11/13/175).

### 2.2 Light microscopy

The freshly collected eyes were used to examine the structures under a light microscope. The eye samples were fixed in 10% neutral buffered formalin solution and subsequently prepared for paraffin sectioning. For general studies four-micrometer serial sections were prepared and stained with hematoxylin–eosin (H&E) stain (31, 32). The Periodic Acid Schiff (PAS) technique was used to stain neutral mucin, reticular fibers, and glycogen (33) and Masson’s trichrome was used to stain collagen fibers (34). The sections were then examined using an Olympus CX31 microscope and photographed using a Canon digital camera (19, 35).

### 2.3 Scanning electron microscopy and morphometric analysis

The *Siganus luridus* fish eye specimens were also used to examine the detailed structure of the cornea, sclera, lens, retina, iris and choroid at the electron microscopy level. At pH of 7.2 and temperature of 4°C, the samples were fixed in a buffer solution consisting of 2% formaldehyde, 1.25% glutaraldehyde, and 0.1 M sodium cacodylate. Following fixation, the samples were washed in 0.1 M sodium cacodylate containing 5% sucrose, treated with tannic acid, and finally dehydrated in ethanol of increasing concentration (15 min in 50, 70, 80, 90, 95, and 100% ethanol). The samples were dried in carbon dioxide, attached to pins with colloidal carbon and coated with gold–palladium in a sputtering device. The samples were examined and photographed with a JEOL JSM-IT200 scanning electron microscope at 15 kV at the Electron Microscope Unit, Faculty of Science, Alexandria University, Egypt (36).

### 2.4 Micrometric analysis

The obtained SEM images were processed using the ImageJ 1.53 k application (National Institutes of Health, United States) to determine the thickness of the retinal layers and the lens dimensions.

## 3 Results

### 3.1 Gross anatomical observations

The body of *Siganus luridus* fish was laterally flattened and had a mainly an ellipsoidal shape. The average length of all of the *Siganus luridus* fish used in this study was 16.20 ± 1.21 cm. Their iris was yellow in color. Their eyes were large; the diameter of the eye at the outer part of the orbit was 11.28 ± 0.80 mm on average and the overall eye width was 8.70 ± 0.09 mm. The horizontal and vertical diameter measurements were the same at 14.20 ± 0.32 mm.

**Figure 1:**
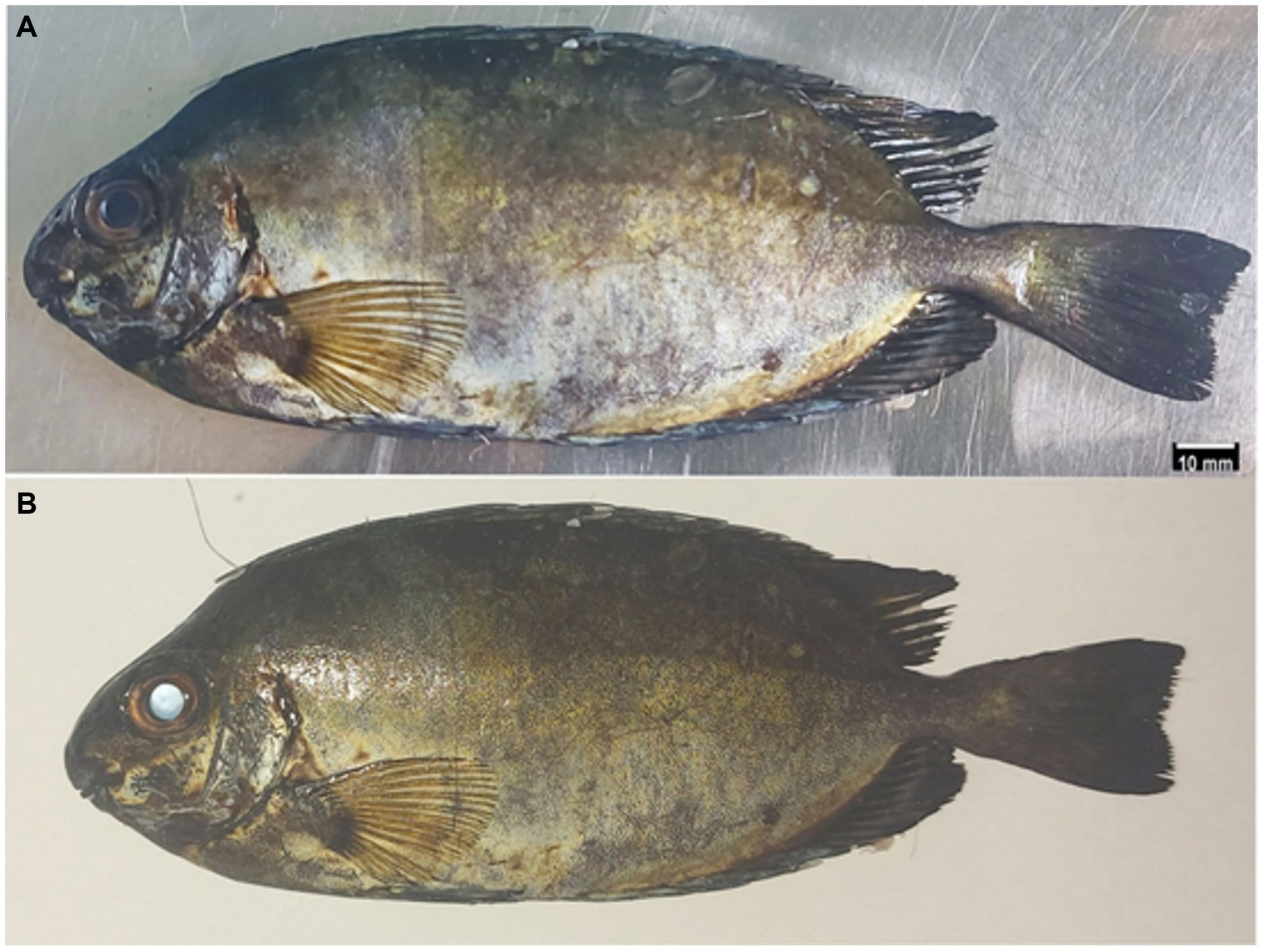
Gross photograph of *Siganus luridus*. The eye had a normal appearance without flash **(A)** and the appearance of the eye-shine with flash **(B)**.

The eyes from the *Siganus luridus* showed a uniform appearance when a flashing light was not shone on it, however it appeared shiny (eye-shine) when a flashing light was applied (Figures 1A,B), indicating the presence of the *tapetum lucidum* (luminous layer), a reflective cell layer behind the retina.

### 3.2 Light and scanning electron microscopic findings in the rostral part of the eye

**Figure 2:**
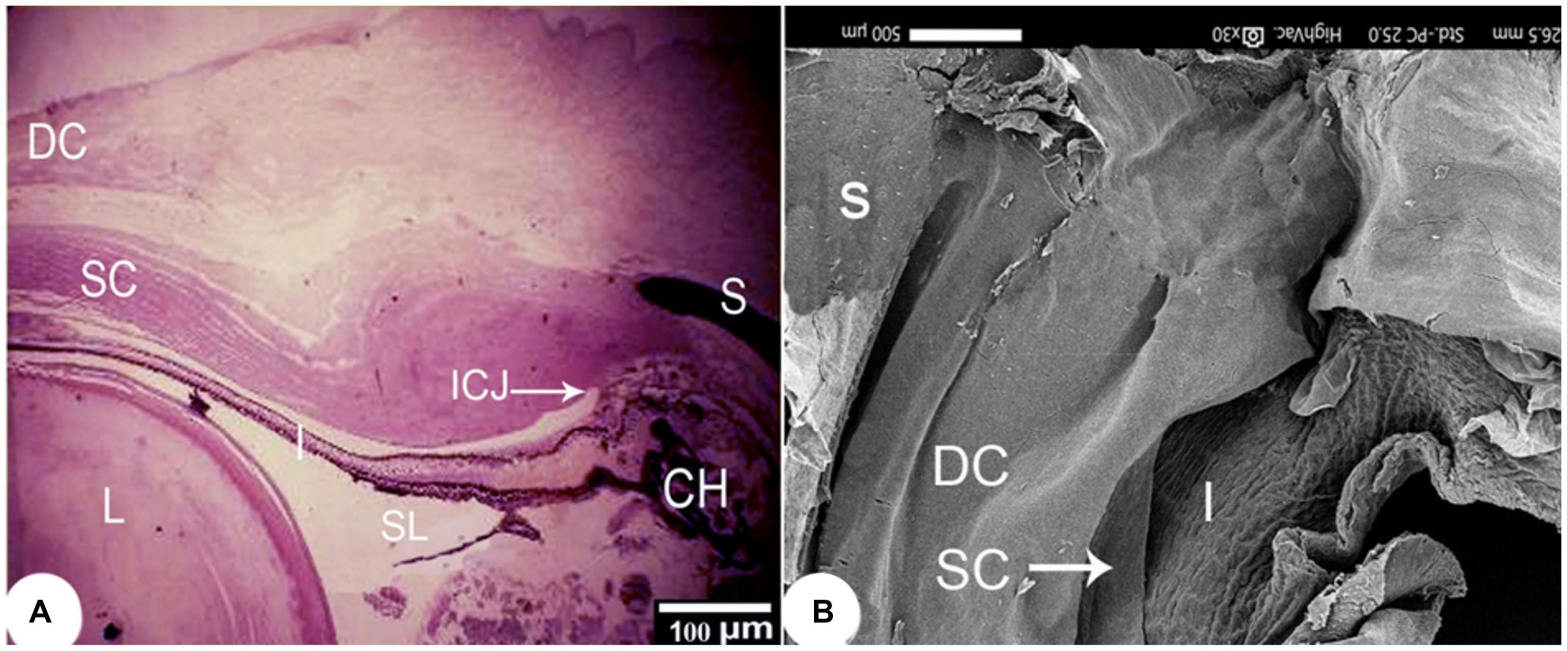
**(A)** Light photomicrograph of the rostral to outer and middle layers of the eye of *Siganus luridus*, H&E staining, magnification x40 and **(B)** SEM of the rostral part of the eye after removal of part of the cornea and lens. The dermal cornea (DC), the scleral cornea (SC), the sclera (S), the iris (I), the suspensory ligament (SL), the iridocorneal junction (ICJ), the choroid (CH), the lens (L).

**Figure 3:**
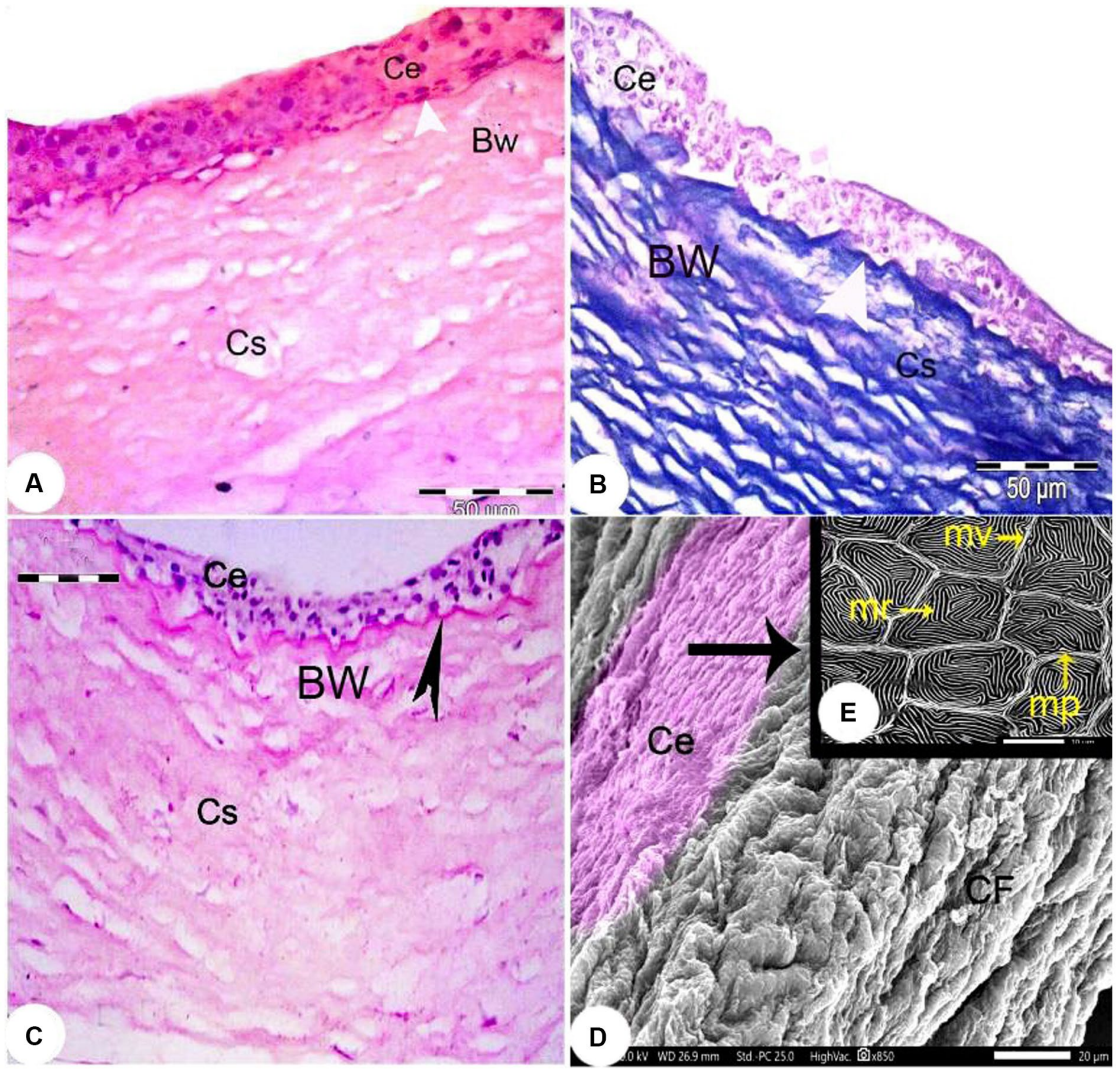
Light photomicrographs of the cornea of *Siganus luridus*. **(A)** H&E staining, magnification x400, **(B)** Trichrome staining, magnification x400, and **(C)** PAS staining with hematoxylin counterstain, magnification x100. The corneal epithelium (Ce), the positive basement membrane of the corneal epithelium (arrowhead), the negative Bowman’s membrane (BW), and the corneal stroma (Cs). **(D)** SEM of the external and sagittal corneal epithelium (Ce) and collagen fibers (*CF*). **(E)** SEM of the *Siganus luridus* fish corneal epithelial cells shows pavement cells with distinct demarcation and microridges (mr), microvilli (mv), and microplicae (mp).

The outermost layer of the *Siganus luridus* eye was divided into a cornea (C) in the anterior and a sclera (S) in the posterior region of the eye. The cornea consisted of both - the dermal cornea (DC) and the scleral cornea (SC). The epithelium of the DC was multilayered and bordered to SC inside the cranium. The cornea consisted of an unpigmented, non-keratinized stratified squamous epithelium, Bowman’s membrane, and the corneal stroma, comprised of several layers of parallel collagen bundles. The suspensory ligament (SL) and the iridocorneal junction (ICJ) were observed (Figures 2A, 3A,C). The rostral part of the cornea consisted of the DC, SC and the iris (I; Figure 2B). The external surface of the cornea resembled a mosaic or a fingerprint with distinct demarcation and showed microridges (mr), microvilli (mv), and microplicae (mp). The sagittal view of the cornea displayed bundles of collagen fibers (Figures 3D,E).

**Figure 4:**
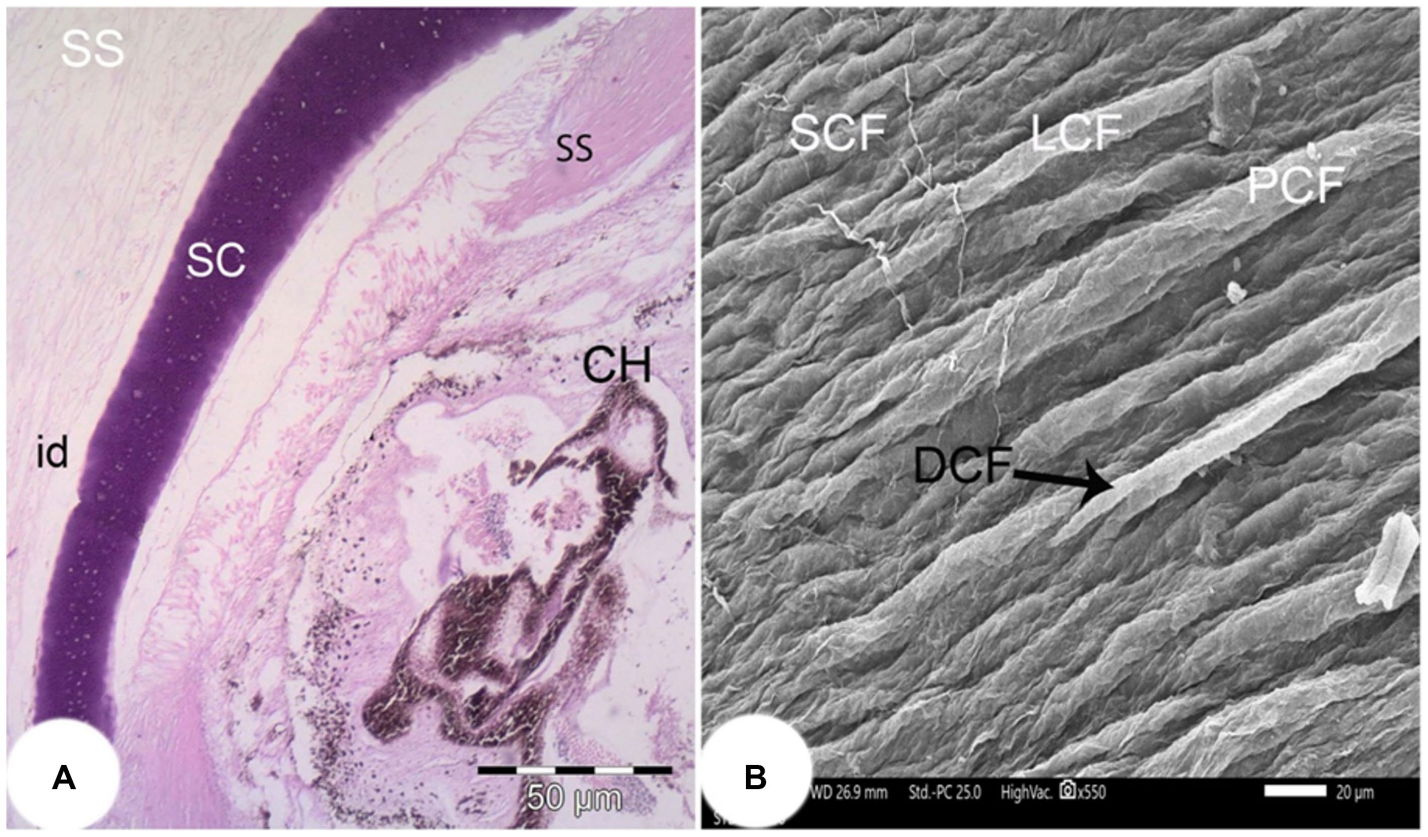
**(A)** Light photomicrograph of the posterior part of the eye of the *Siganus luridus* (H&E staining, magnification x100) showing the iridescent layer (id), the sclera stroma (SS), the scleral cartilage (SC), and the choroid (CH). **(B)** SEM of the outer region of the sclera of *Siganus luridus* fish explaining parallel collagen fibers (PCF), short collagen fibers (SCF), long collagen fibers (LCF), and collagen fiber divergence (DCF).

The corneal epithelium (Ce) basement membrane was PAS-positive. In contrast, the Bowman’s membrane (BW) was PAS-negative (Figure 3B). The wall of the posterior part of the eye consisted of a thick fibrous sclera (S) with an outer covering of hyaline cartilage. An iridescent layer was located between the anterior and posterior scleral stroma (Figure 4A). The superficial layer of the sclera contained both short and large collagen fibers (LCF). These LCFs appeared to run parallel to each other, branched into two branches, and were interwoven with the underlying structures. In contrast, the short collagen fibers (SCFs) had differing sized diameters and were mainly intertwined with each other. Collagen fiber divergence (DCF) was observed (Figure 4B).

### 3.3 Light and scanning electron microscopic findings in the lens

**Figure 5:**
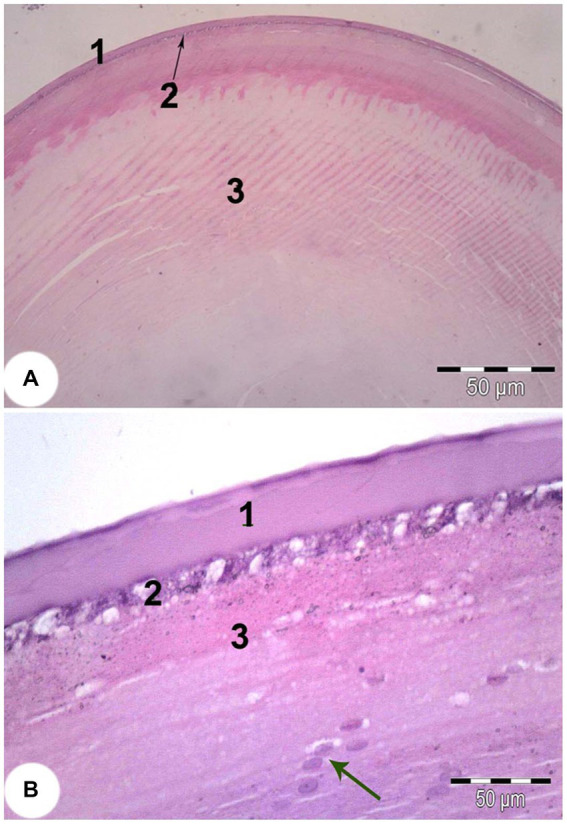
Light microscope image of the lens of *Siganus luridus* (H&E staining, magnification **(A)** x100) and **(B)** x400). (1) simple cuboidal epithelium, (2) lens fibers, (3) lens fibers, cell nuclei (arrow).

The lens of the eye was completely round and protruded into the aqueous chamber. It consisted of three layers: an outer acellular sheath, an underlying simple cuboidal epithelial layer, and an inner fibrous layer. At the equator of the lens the cuboidal epithelium changed into a columnar epithelium. The cell nuclei in the newly formed lens fibers were visible (Figures 5A,B).

**Figure 6:**
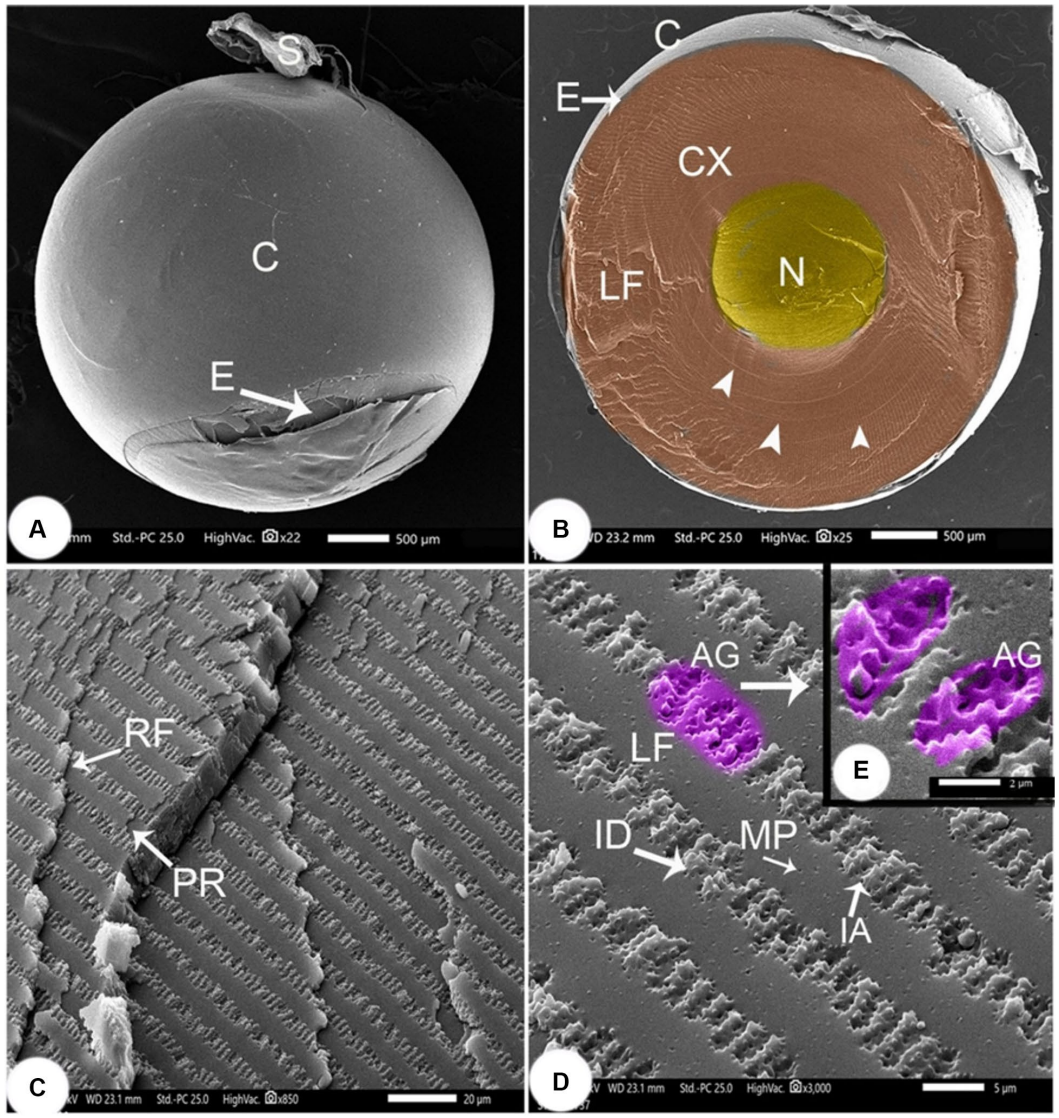
SEM image of the lens of *Siganus luridus* **(A)** SEM image of the spherical lens, **(B)** SEM image of a sagittal section of the eye, **(C)** SEM image showing the cortex with layers of rows of lens fibers, **(D)** SEM image showing a row of lens fibers, and **(E)** SEM image showing interdigitations between the lens fibers. Lens capsule (C), suspensory ligament (S), lens epithelium (E), cortex (CX), embryonic nucleus (N), concentric circles (arrowheads), lens fibers (LF), row of lens fibers (RF), parallel of lens fibers (PR), ball and socket interdigitations (ID), (AG) arched groove, (IA) irregular arches and micropores (MP).

The remnant of the suspensory ligament (SL; Figure 6A) diameter was 3647.80 ± 10.80 μm. Its dorsoventral diameter was 3,726 ± 2.25 μm. A capsule of collagen fibers surrounded the lens (L) and was 24.51 ± 0.05 μm thick. Lens epithelium (LE) cells were found beneath the capsule. The central area of the lens contained spherical nuclei (N), which were 1,283 ± 3.12 μm wide and a cortex.

The lens cortex had concentric circles in the center and consisted of lens fibers (LF) with a width of 8.56 ± 0.27 μm, and a height of 0.66 ± 0.048 μm. Straight embryonic fibers ran along the anterior–posterior axis (Figure 6B). The densely packed fibers that formed the continuous layers appeared rectangular, revealing the arrangement of cortical lens cells in rows. The lens fibers ran parallel in consecutive rows (Figure 6C), and adjacent rows were connected through junctions, called interdigitations (ID). Spherical connecting pores were observed on the lens fibers. The ID were spherical with irregularly curved (IA) protrusions that were 2.93 ± 0.13 μm long and 1.02 ± 0.04 μm wide. The arched lens fiber projections, each with a peripheral tooth, were interlocked with the adjacent lens fiber at the peripheral perforated arched groove with 6–8 microholes. The gap was very small, and continuous interlocking was observed (Figures 6D,E).

### 3.4 Light and scanning electron microscopic findings in the middle and posterior parts of the eye

**Figure 7:**
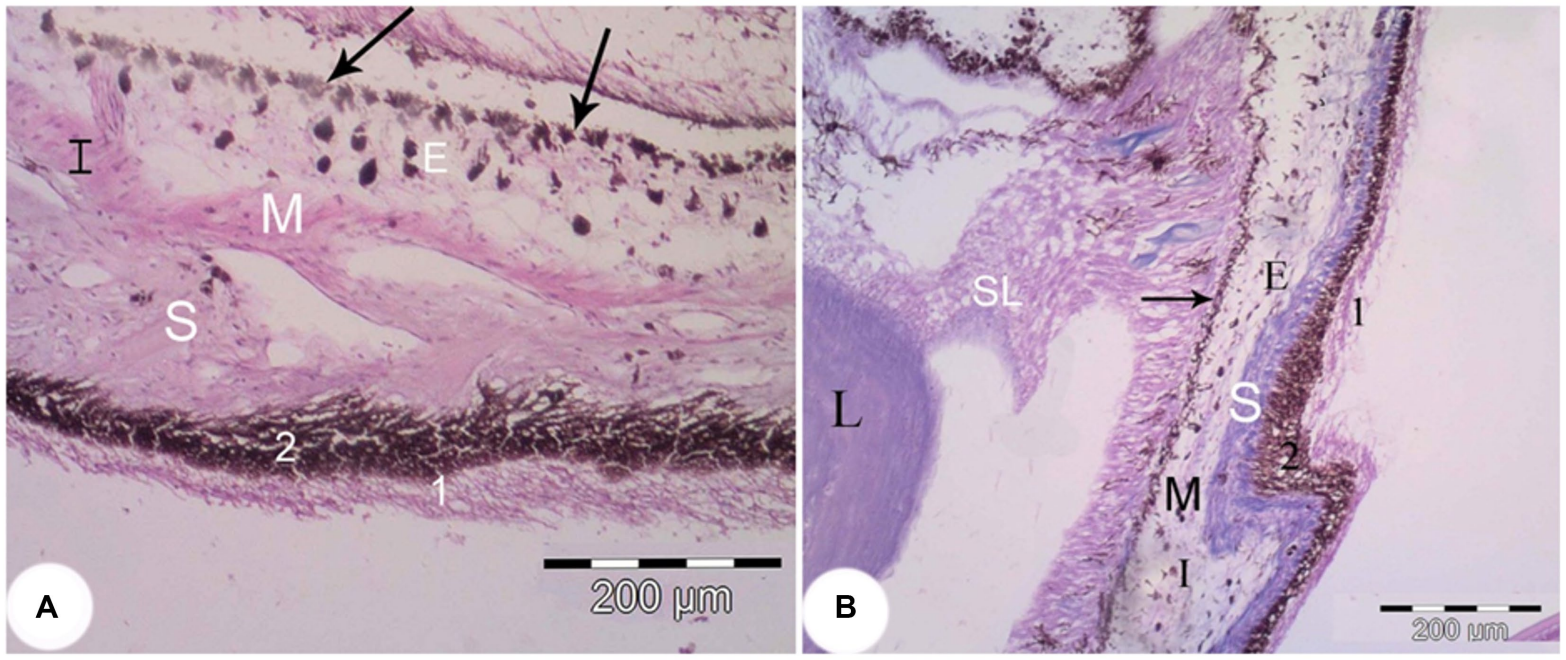
The iris of the *Siganus luridus*. **(A)** A light photomicrograph (H&E staining, magnifica-tion x400). **(B)** Trichrome staining, magnification x100. The iris (I), the posterior part of the iris (arrow), smooth muscle (M), stroma (S), melanocytes (E), non-pigmented simple cuboidal epi-thelium (1), pigmented simple cuboidal epithelium (2), suspensory ligament (SL), and lens (L).

The middle and posterior layers of the eye contained the choroid and the iris in addition to the lens. The iris consisted of an anterior part, a posterior part, and a stroma. The anterior part consisted of a discontinuous layer of loose connective tissue and melanocytes. The stroma occupied a large part of the iris and consisted of loose connective tissue, smooth muscle, melanocytes, and blood vessels. The posterior part of the iris showed a double layer of cuboidal epithelium, an outer non-pigmented layer and an inner pigmented layer (Figures 7A,B).

**Figure 8:**
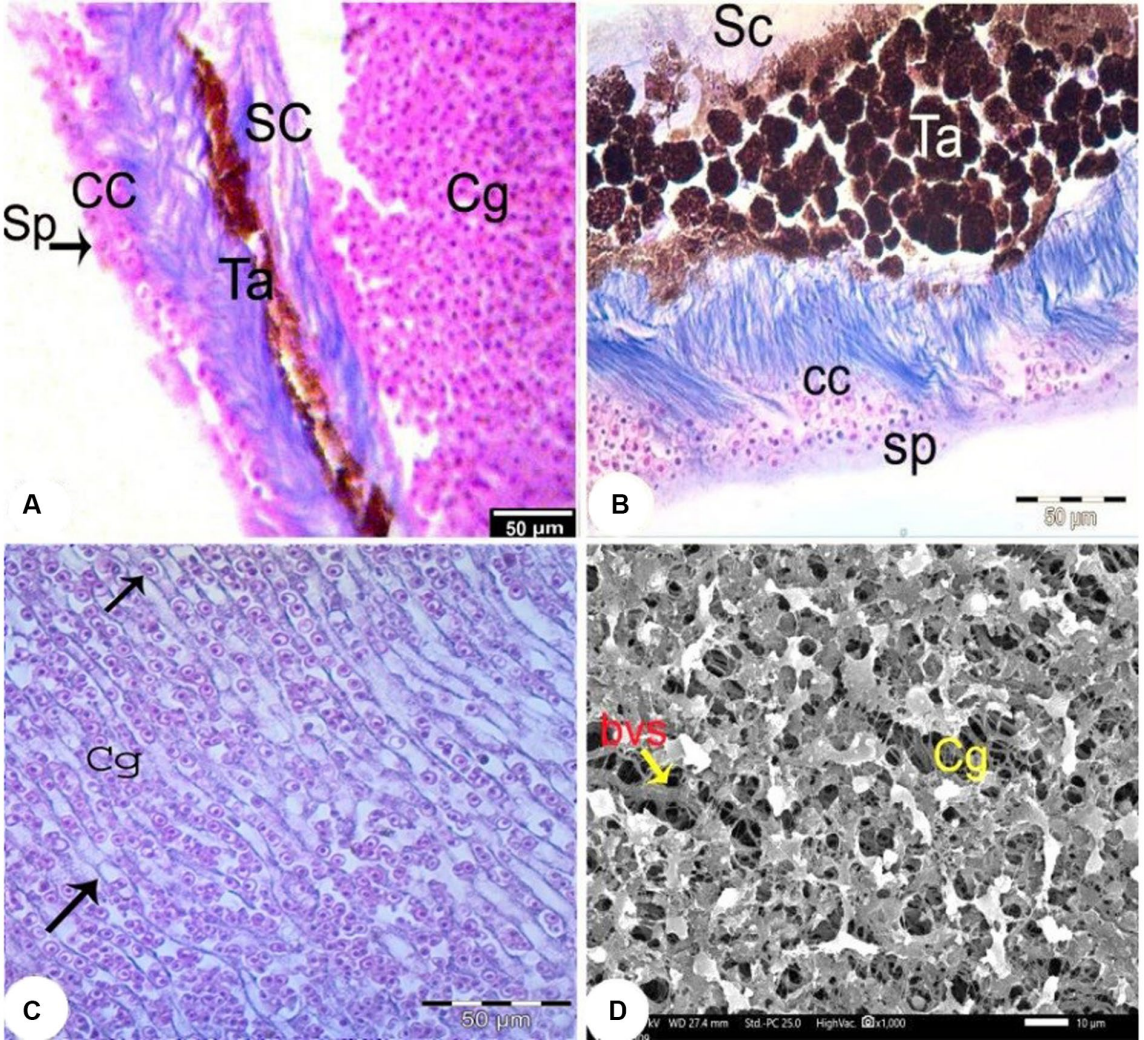
Light photomicrographs of the choroid (Trichrome staining, magnification x400) **(A–C)** showing the choroidal gland (Cg), the suprachoroidal layer (SC), the fibrous substantia propria layer (Sb), the choriocapillary layer (CC) and the separating layer (Sp) and blood capillaries containing nucleated RBC (arrow). **(D)** SEM of the choroidal gland (Cg) with widely distributed blood vessels (bvs).

The choroid was divided into four layers: the suprachoroidal layer, the tapetal layer, the choriocapillary layer, and the separator layer (Figure 8A). The suprachoroidal layer consisted of loose connective tissue in which numerous melanocytes and blood vessels (bvs) were embedded. The *tapetum lucidum* layer consisted of dense choroidal tissue infiltrated with numerous melanocytes and blood vessels (Figures 8B,C). Numerous small blood vessels were detected in the choroidal gland and formed a *rete mirabile* between the sclera and the pigmented retinal epithelium. SEM examination of the choroidal gland revealed pores with densely packed blood vessels (Figure 8D).

**Figure 9:**
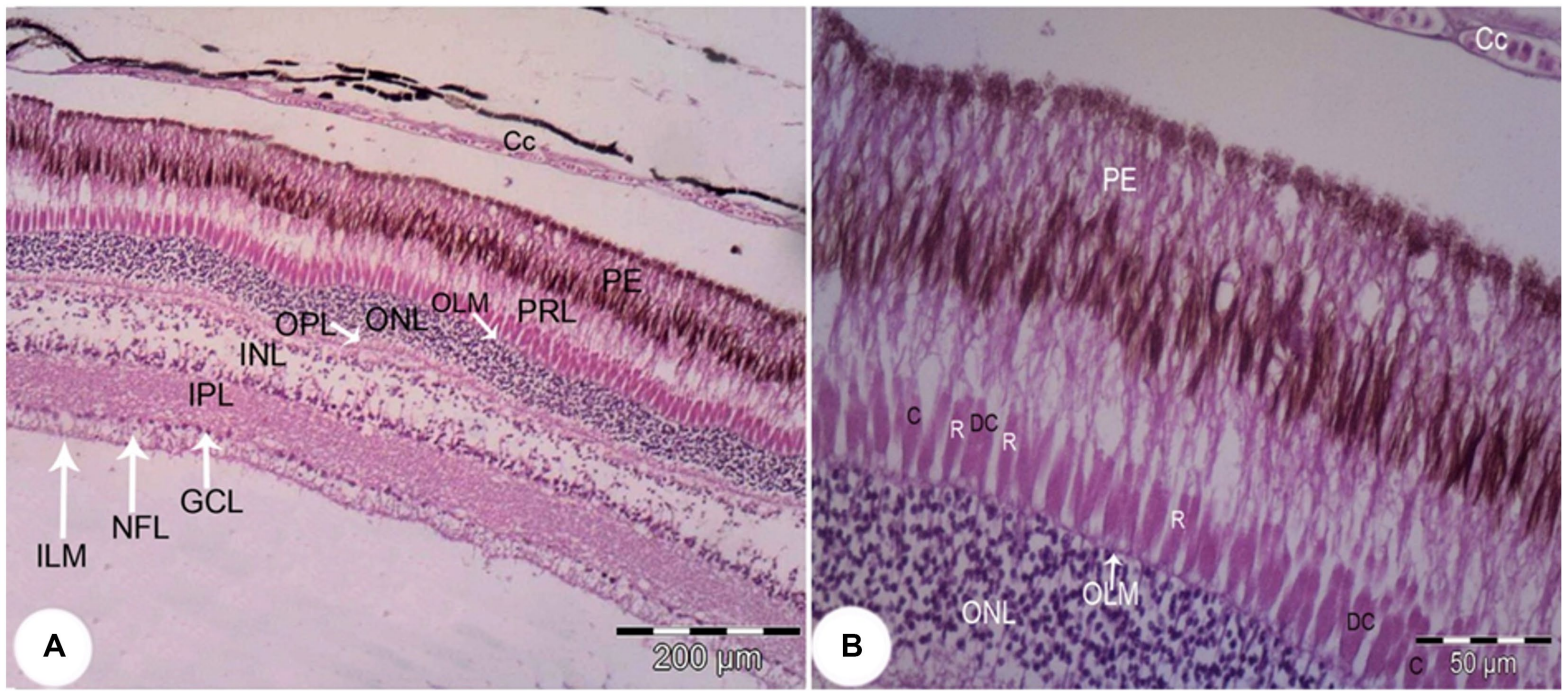
Light photomicrograph of the retina of *Siganus luridus*: H&E, staining, magnification **(A)** x100), **(B)** x400. Choriocapillary (CC), pigmented epithelium (PE), photoreceptor layer (PRL), outer limiting membrane (OLM), outer nuclear layer (ONL), outer plexiform layer (OPL), inner nuclear layer (INL), inner plexiform layer (IPL), ganglionic cell layer (GCL), nerve fiber layer (NFL), inner limiting membrane (ILM), rods (R), cones (C), and double cones (DC).

The retina consisted of 10 layers, which were arranged from outside through to the inside of the eye as: a pigmented epithelial layer (PE), photoreceptor layer (rods and cones; PRL), outer limiting membrane (OLM), outer nuclear layer (ONL), outer plexiform layer (OPL), inner nuclear layer (INL), inner plexiform layer (PL), ganglion cell layer (GCL), nerve fiber layer (NFL), and an inner limiting membrane (ILM; Figure 9A). The photoreceptor layer was composed of rods, single cones, and double cones (Figure 9B). The average thickness of the retina in the middle of the globe was 355.61 ± 1.34 μm. The photoreceptor layer was the thickest of the layers (averaging 67.87 ± 3.33 μm), followed by the pigmented epithelium (averaging 62.98 ± 2.17 μm) and the inner plexiform layer (57.67 ± 0.96 μm), which maximized the connectivity activity of retinal neurons. However, the inner nuclear layer and the optic nerve layer were the thinnest. ONL/INL was measured 0.79 ± 0.08 μm, which corresponds to diurnal activity of *Siganus luridus*.

The greatest thickness was observed in the photoreceptor layer (PRL), followed by the pigmented epithelium (PE), the inner plexiform layer (IPL), the inner nuclear layer (INL), and then the outer nuclear layer (ONL). In contrast, the thinnest layers were the outer limiting membrane (OLM) and the inner limiting membrane (ILM), followed by the ganglion cell layer (GCL), the outer plexiform layer (OPL), and the nerve fiber layer (NFL).

**Figure 10:**
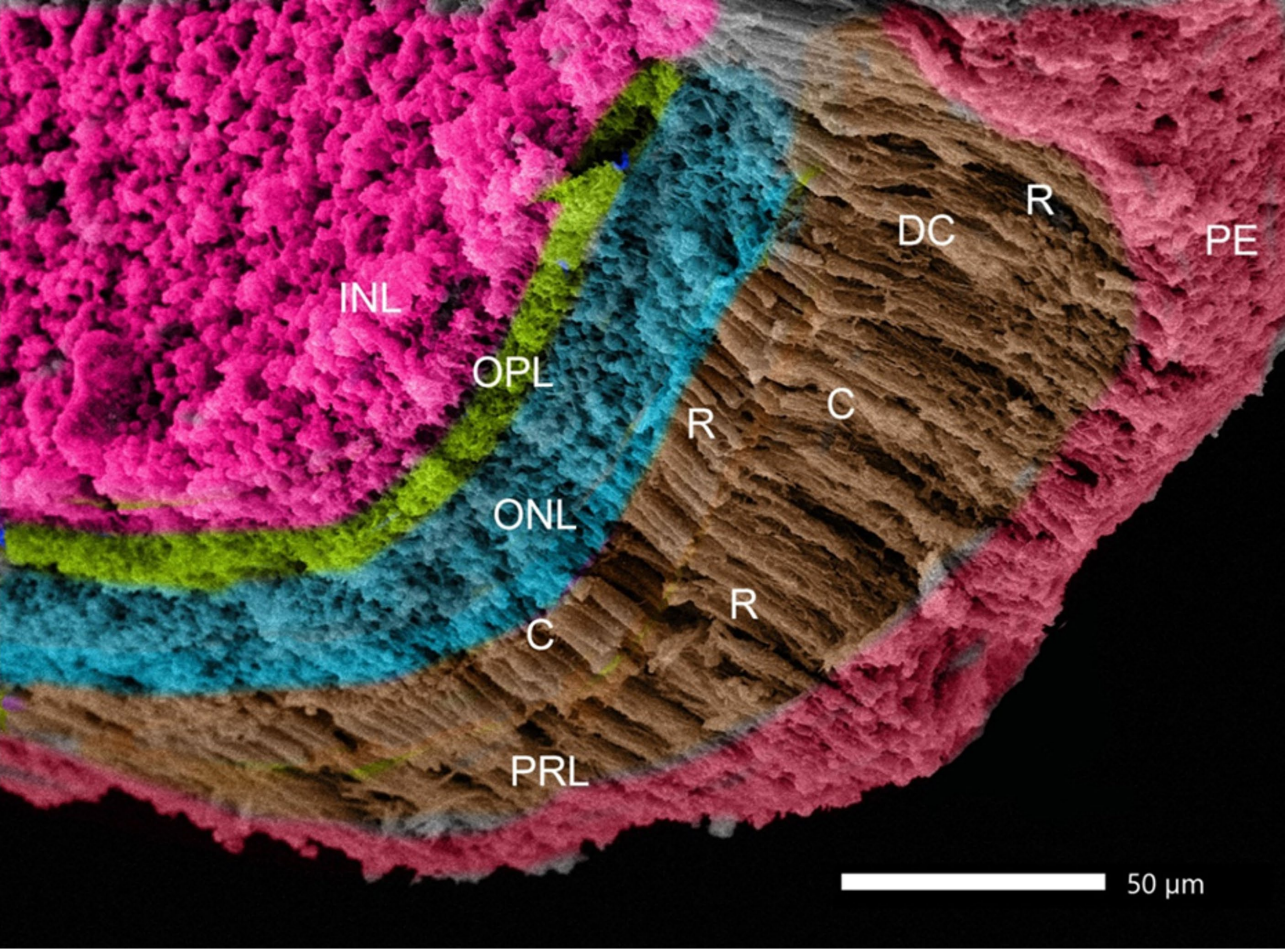
SEM image of the retina of *Siganus luridus*: is showing the following layers: retinal pigment epithelium (PE), photoreceptor layer (PRL), outer nuclear layer (ONL), outer plexiform layer (OPL), inner nuclear layer (INL), rods (R), cones (C), and double cones (DC).

Rods, single cones, and double cones characterized the photoreceptor layer of the retina. The inner and outer nuclear layers were distinguished by a small sphere-like structure (Figure 10).

**Figure 11:**
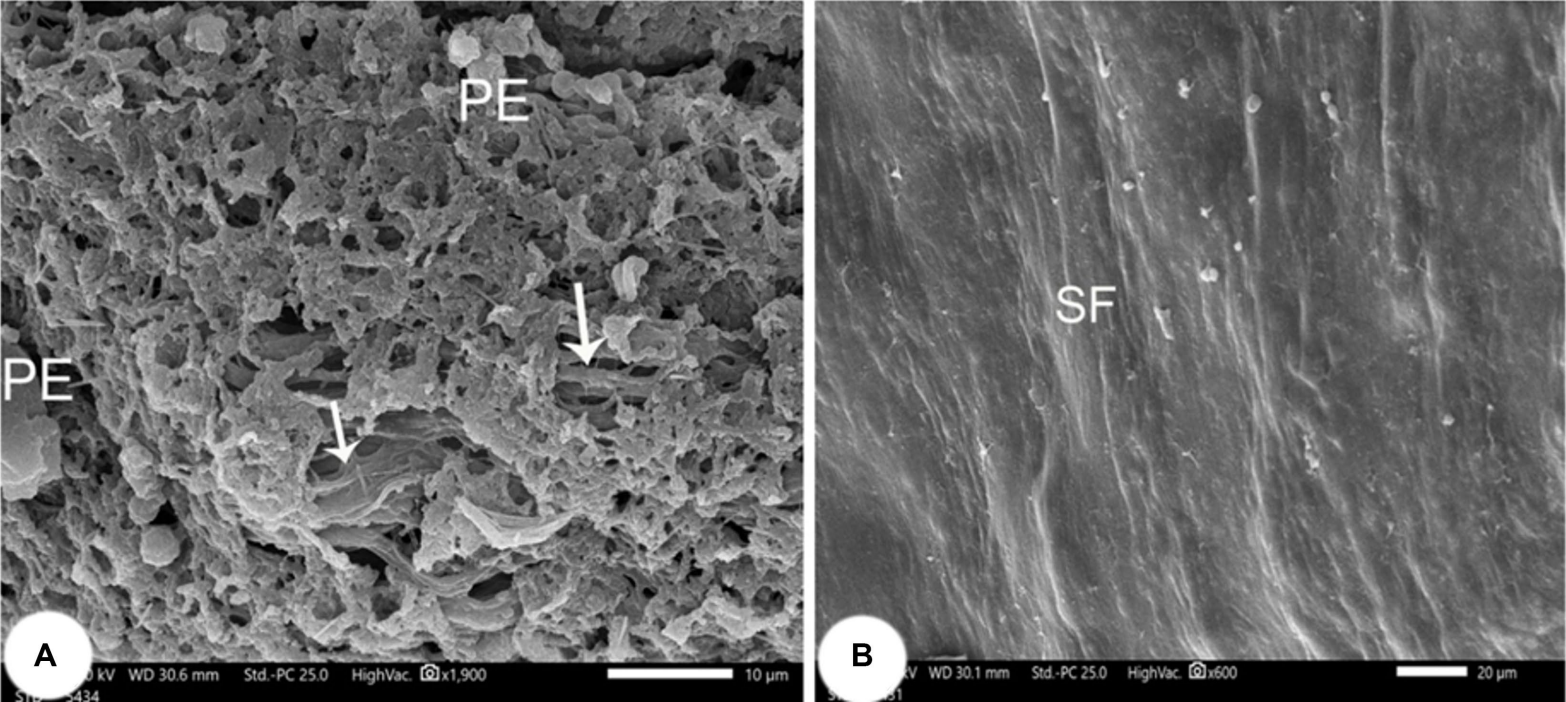
Retina of *Siganus luridus.* **(A)** SEM of the apical surface of the pigment epithelium (PE) and the apical processes interdigitated with the outer segments of rods and cones. **(B)** SEM of the inner surface of the retinal epithelium, which appears in the form of small folds (SF).

The pigmented layer had apical processes that interdigitated with the outer segments of the rods and cones (Figure 11A). The inner limiting membrane was smooth with minor folds (SF; Figure 11B).

**Figure 12:**
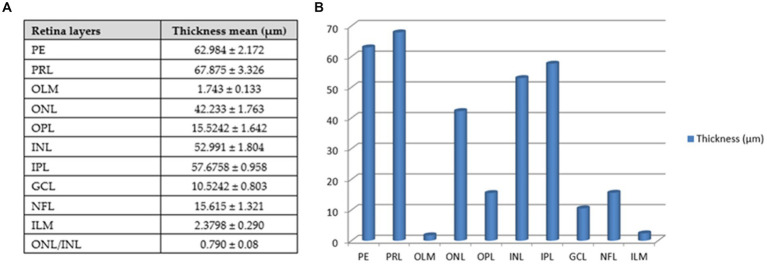
Thickness of retinal layers of *Siganus luridus*. **(A)** A Table with the mean thickness of the retinal layers of *Siganus luridus* fish, **(B)** A graph showing the differences in the mean thickness of the retinal layers of *Siganus luridus* fish. (PE) pigmented epithelium, (PRL) photoreceptor layer, (OLM) outer limiting membrane, (ONL) outer nuclear layer, (OPL) outer plexiform layer, (INL) inner limiting membrane, inner plexiform layer (IPL), (GCL) ganglion cell layer, (NFL) nerve fiber layer, (ILM), and (ONL) outer nu-clear layer /(INL) inner nuclear layer.

The average thickness of the retinal layers of *Siganus luridus* was determined from the SEM images using Image J software (Figure 12).

## 4 Discussion

*Siganus luridus*, or dusky spinefoot, is vital for coral reefs as it controls algal growth, maintaining ecosystem balance. Its population indicates reef health, reflecting environmental conditions. Understanding its structure, behavior, and interactions, especially its feeding habits and vision, is crucial for marine biodiversity (11, 12). Studying *Siganus luridus* aids in understanding marine species adaptation and behavior, supporting coral reef conservation. Economically important, it contributes to local fisheries and food security. Understanding its population dynamics is crucial for sustainable fishing and the aquarium trade (11, 12).

The effects of the aquatic environment on vision, including light conditions, depth and water quality, altered visual adaptation or some structures that improve sensitivity, such as pigment, tapeta and photoreceptor layers (37). The morphometry of eye size has been related to feeding habits and activity time (36). The percentage of the eye diameter to the length of the fish is reported to be 12–13% in nocturnal fish species, while it was less than 10% in diurnal fish species (38). In *Siganus luridus* it was 8.70 ± 0.09%, which is consistent with the herbivore nature of the fish.

The eye size of carnivores was relatively or absolutely larger than that of herbivores. Diurnal planktivores and nocturnal species with small body size maximized their vision by having relatively large eyes. The planktivores generally had relatively larger eyes than the large-bodied species. This presumably compensates for the limitations in vision caused by small body size. Nocturnal and crepuscular species all had relatively or absolutely large eyes, which not only maximize visual acuity but also increase the sensitivity of the visual system to point sources of light (e.g., bioluminescence) (36, 39).

The cornea, the initial gateway in the visual process, possesses maximal light transmission and unique light-filtering structures, such as epithelial microprojections and stromal structures (40). We observed the sclera and dermal cornea in *Siganus luridu* is similar to the rabbitfish eye (41). The corneal stroma was found to consist of collagen fibers, while the simple corneal endothelium was composed of nonkeratinized squamous epithelium (14, 40). The corneal epithelium of *Siganus luridus* was multilayered and bordered the integument of the head. The corneal epithelium was continuous with the conjunctiva and the skin. This result has been found in all aquatic vertebrates (40). Our study revealed that the cornea of *Siganus luridus* consisted of an unpigmented, non-keratinized stratified squamous epithelium, Bowman’s membrane, and the corneal stroma. At the same time, the corneal epithelium of *Malapterurus electricus* and the European eel (*Anguilla Anguilla*) exhibit a specific distribution of dense pigmentation in the epithelium and dispersed ellipsoid bodies in the upper stromal layers (42). The corneal epithelium basement membrane in the present study was PAS-positive, indicating the presence of glycoproteins (43). The anterior cornea is multilayered and contains glycoproteins that protect the eye and act as a barrier to limit hydration and maintain transparency. The epithelium of the basement membrane was thin and mainly present in shallow water fish, and thicker in deep water fish (40). Using SEM, the dorsal surface of the cornea appeared as mosaics or fingerlike structures with marked demarcation and carried microridges, microvilli, and microplicae. This structure increases the surface area and strength of the cornea by adapting to high-osmolarity seawater, which increases the cell surface area and improves the supply of oxygen and nutrients to the cornea (43–45).

An iridescent layer was observed on the cornea of *Siganus luridus*. This feature has also been observed in other teleost fishes, such as *Pomatoschistus minutus* (27), *Platichthys flesus* (46), and *Lepidoglaxias salamandroides* (47). Functions of the iridescent layer include birefringence, a color filter, a polarizing filter, camouflage or display, and enhancement or suppression of reflection (48).

Many fish have an iridescent layer that becomes visible when the eye is illuminated from above. Iridescence is particularly common in shallow-water teleosts that live between rocks or on the bottom. Iridescence occurs when light passes through a regular multilayer stack, with the thickness of each layer being of the order of a quarter of the wavelength of visible light. However, the components of these layers are different (49).

Scleral cartilages are found in teleosts (25) and two scleral cartilages have been discovered in salmon and zebrafish (50, 51). In our investigations, scleral cartilages were found, but no scleral ossicles were observed (14). The difference in the morphology of the eye skeleton within the vertebrates is probably related to selection pressure in the course of evolution. However, this pressure is probably different in teleosts and reptiles, as the intraocular pressure in teleosts does not change during the process of visual accommodation (51). It is possible that the stronger mechanical support provided by a deep cartilaginous element is advantageous in deeper, benthic habitats where water pressure is higher, whereas a lighter, narrower ring is advantageous in pelagic habitats where changes in velocity or depth cause ossification of the cartilage to form ossicles (51, 52). Benthic fish generally do not have scleral ossicles (51) and are known to generate increased intraocular pressure through fluid regulation in the choroid to withstand the ambient pressure in benthic environments (53). The reason for the greater depth of the scleral cartilage in teleosts living in benthic environments may therefore differ from that of cave-dwelling species and is likely to be complex. Further studies are needed to fully understand the evolutionary history of this element in teleosts.

The lens of *Siganus luridus* consisted of three layers: an outer acellular sheath, an underlying simple cuboidal epithelial cell layer, and an innermost fibrous layer. It was possible to see the nuclei of the newly formed lens fibers. In the juvenile Queen Danio, *Devario regina*, the lens consisted of sheaths of non-cellular transparent material, a monolayer of epithelial cells and lens fibers (43). All fish species showed the same structural organization of the lenticular fibers. The lens contained a monolayer of anterior epithelial cells and concentric layers of differentiated fiber cells from the periphery toward the center. A similar pattern of fiber cell ribbon shapes and interdigitating knobs was described by Zigman in sharks and skates (54). In the shallow and middle water depths, there was sufficient light for the fish to recognize their food. In dark furrows or caves, there is no outflow of daylight, so fish have evolved numerous features of the visual system that allow them to behave under different types of light conditions by increasing the thickness of the entire retinal cell layer and adapting both lens and corneal architecture (42).

In *Siganus luridus*, the choroidal gland (choroids *rete mirabile*) was visible between the sclera and the pigment epithelium. The same result was also found in many teleost fish including, *Gambusia schoelleri* (55), *Epinephelus fuscoguttatus* (56), and in the rabbitfish (*Siganus rivulatus*) (41). *Anguilla anguilla* lacks the choroid gland (57). The function of the choroidal gland is to maintain a high oxygen pressure at the retina and to facilitate transport from the choriocapillaris to the retinal pigment epithelium (57). It may also function as a cushion against compression of the eyeball. In our study, the choroid of *Siganus luridus* was found to have a *tapetum lucidum* (light-reflecting layer) that may help at nocturnal activity such as feeding. In several teleost species examined by conventional light and fluorescence microscopy, the eye shine phenomenon was associated with the presence of a reflective surface in the eye, the *tapetum lucidum* (58). This layer was also observed in the choroid of *Siganus javus* (41). Fish with a *tapetum lucidum* are usually associated with dimly lit environments, i.e., the deep sea, muddy rivers and turbid water, or with nocturnal activity. In fact, fish with tapeta are found at intermediate water depth, e.g., species of *Chlorophthalmus*, *Polymixia* and *Epigonus* and non-herbivorous fishes (58). The choroidal tapetum, consisting of reflective cells that lie immediately outside the choriocapillaris, is formed in a few primitive teleosts. More commonly, the *tapetum lucidum* is an integral part of the pigment epithelium in primitive teleosts (it lies within the processes of the pigmented epithelial cells) (59). African catfish have a *taptum lucidum* and eyes that are adapted not only to nocturnal but also to daylight (19, 60). However, it was interesting to observe the *tapetum lucidum* in *Siganus luridus*, as it is a fish, that lives in shallower waters and is herbivorous. Fish living in shallow waters generally have less developed *tapetum lucidum* compared to their deep-water counterparts.

Regarding the relationship between the photoreceptors and their environment, our results showed that the photoreceptor layer included rods, single cones and double cones. The photoreceptor layer was the thickest, followed by the pigmented epithelium. The ONL/INL was measured 0.79 ± 0.08 μm indicating our *Siganus luridus* exibits diurnal activity. In the ONL/INL ratio, *Oreochromis*. *niloticus* had the lowest average ratio (0.53 ± 0.07), indicating diurnal activity. This ratio increased in *Bagrus bajad* (2.80 ± 0.83), *Chrysithys auratus* (1.70 ± 0.25) and *Clarias gariepinus* (1.50–0.53), suggesting nocturnal activity. The average ratio of ONL/INL increased in *Bagrus bajad*, *Chrysithys auratus* and *Clarias gariepinus*, suggesting nocturnal habits and reflecting the structural pattern of photoreceptors. *Nile tilapia* usually feed during the day, which indicates that they show a behavioral response to light as the main factor for feeding activity, similar to trout and salmon (61).

The importance of the study is reflected in the finding that *Siganus luridus* is a herbivore, that feeds not only during the day but also at night.

## Data Availability

The raw data supporting the conclusions of this article will be made available by the authors, without undue reservation.
